# Special Issue “Novel Research about Biomechanics and Biomaterials Used in Hip, Knee and Related Joints”

**DOI:** 10.3390/ma14092222

**Published:** 2021-04-26

**Authors:** Jan Philippe Kretzer, Catherine Van Der Straeten

**Affiliations:** 1 Laboratory of Biomechanics and Implant Research, Clinic for Orthopedics and Trauma Surgery, Heidelberg University Hospital, 69118 Heidelberg, Germany; 2 Health Innovation and Research Institute, Ghent University Hospital, C. Heymanslaan 10, 9000 Gent, Belgium; catherine.vanderstraeten@ugent.be

Joint replacement is a very successful medical treatment. However, the survivorship of hip, knee, shoulder, and other implants is variable depending on the material and the loading conditions. The degradation of materials, but also the immune response against degradation products or an altered tissue loading condition as well as infections remain key factors of their failure.

Current research in biomechanics and biomaterials is trying to overcome the existing limitations. This includes new implant designs and materials, bearings concepts and tribology, kinematical concepts, surgical techniques, and anti-inflammatory and infection prevention strategies. A careful evaluation of new materials and concepts is required in order to fully assess the strengths and weaknesses and improve the quality and outcomes of joint replacements. Therefore, extensive research and clinical trials are essential.

The main aspects that are addressed in this Special Issue are related to new materials, the design and manufacturing considerations of implants [1,2,3,4,5,6,7,8,9,10], implant wear and its potential clinical consequences [11,12,13,14,15,16], implant fixation [17,18,19], infection-related material aspects [20,21] and taper-related research topics [22,23,24].

This Special Issue gives an overview of the ongoing research in these fields. Contributions are solicited from researchers working in the fields of biomechanics, biomaterials, and bio- and tissue-engineering.

We hope you enjoy reading this book.
